# Reply to Spiess, B.D.; Houdijk, W.P.M. Comment on “Saner et al. The Yin and the Yang of Hemostasis in End-Stage Liver Disease. *J. Clin. Med.* 2023, *12*, 5759”

**DOI:** 10.3390/jcm13061668

**Published:** 2024-03-14

**Authors:** Fuat H. Saner, Ecaterina Scarlatescu, Dieter C. Breoring, Dmitri Bezinover

**Affiliations:** 1 King Faisal Specialist Hospital & Research Center, Organ Transplant Center of Excellence, Riyadh 11564, Saudi Arabia; dbroering@kfshrc.edu.sa; 2 Department of Anesthesia and Intensive Care Medicine III, Fundeni Clinical Institute, 022328 Bucharest, Romania; katyscarlatescu@yahoo.com; 3 Anesthesia and Intensive Care Department, University of Medicine and Pharmacy “Carol Davila”, 050474 Bucharest, Romania; 4 Penn Anesthesiology and Critical Care HUP, Hospital of the University of Pennsylvania, Philadelphia, PA 19104, USA; dbezinover@gmail.com

Thank you for your insightful comments on our article [1] and for bringing attention to the Quantra^®^ system. We appreciate your emphasis on the importance of viscoelastic tests (VETs) in the management of coagulation disorders, particularly end-stage liver disease.

While there are some strong recommendations in place [2,3,4], it still cannot be said that VETs are now standard. There are still many physicians who are very reluctant to use VETs.

We agree that the Quantra^®^ system, which uses ultrasound resonance to directly measure the physical strength of blood as it changes from a liquid to a gel, is a significant development in the field of coagulation analysis [2,5]. It provides a rapid, cartridge-based, point-of-care (POC) VET system that has shown promising results in various clinical settings, including for liver transplantation [2,5].

However, we would like to clarify that our focus on rotational thromboelastometry (ROTEM) and/or TEG as a gold standard in our review is based on its extensive validation in numerous clinical studies and its widespread use in clinical practice [6]. ROTEM provides a comprehensive depiction of the coagulation process and has been shown to significantly reduce the amount of transfusion without an increase in bleeding events in patients with end-stage liver disease [6].

Quantra offers the ability to evaluate the clot lysis index, assess fibrinolysis, and determine whether to add tranexamic acid to the starter agent. It also allows for the differentiation of contributions made by fibrinogen and platelets [7]. Similarly, Rotem provides these capabilities. With ROTEM, fibrinolysis is quantified by clot lysis indices (which represent residual clot amplitude 30, 45, and 60 min after the clotting time as a percentage of maximum clot firmness (MCF)) or by maximum lysis (ML); the difference between MCF and the lowest amplitude during runtime is also expressed as a percentage of MCF. Additionally, fibrinolysis can be detected with ROTEM by comparing the results from the extrinsically activated channel, EXTEM, with APTEM, which uses extrinsic activation and contains an antifibrinolytic agent (tranexamic acid).

Additionally, by comparing the FIBTEM channel, where platelets are neutralized with cytochalasin D, with the EXTEM channel, which displays the combined clot firmness of platelets and fibrinogen, ascertaining whether a patient requires fibrinogen or platelets becomes straightforward.

While the Quantra^®^ system offers a unique approach to measuring clot strength, it is important to note that it indirectly measures clot strength using ultrasound, as opposed to mechanical measurements provided by ROTEM [8]. Both systems have their strengths and limitations, and the choice between them should be guided by the specific clinical context and the available evidence.

We appreciate your suggestion to include a discussion of the Quantra^®^ system in our review. We agree that it is important to keep abreast of all available technologies and to continually reassess the definition of “standard” as new evidence emerges.

Although PubMed lists 50 publications related to Quanta, there are 2178 citations available for ROTEM in the same database (7 December 2023; 17:10 CET).

We look forward to seeing more research on the Quantra^®^ system and its potential role in managing coagulation disorders associated with end-stage liver disease.

## References

[1] Spiess B.D., Houdijk W.P.M. (2024). Comment on Saner et al. The Yin and the Yang of Hemostasis in End-Stage Liver Disease. *J. Clin. Med.* 2023, *12*, 5759. J. Clin. Med..

[2] Hartmann J., Hermelin D., Levy J.H. (2023). Viscoelastic testing: An illustrated review of technology and clinical applications. Res. Pract. Thromb. Haemost..

[3] Rossaint R., Afshari A., Bouillon B., Cerny V., Cimpoesu D., Curry N., Duranteau J., Filipescu D., Grottke O., Grønlykke L. (2023). The European guideline on management of major bleeding and coagulopathy following trauma: Sixth edition. Crit. Care.

[4] Wikkelso A., Wetterslev J., Moller A.M., Afshari A. (2016). Thromboelastography (TEG) or thromboelastometry (ROTEM) to monitor haemostatic treatment versus usual care in adults or children with bleeding. Cochrane Database Syst. Rev..

[5] Baulig W., Akbas S., Schütt P.K., Keul W., Jovic M., Berdat P., von Felten S., Steigmiller K., Ganter M.T., Theusinger O.M. (2021). Comparison of the resonance sonorheometry based Quantra® system with rotational thromboelastometry ROTEM® sigma in cardiac surgery—A prospective observational study. BMC Anesthesiol..

[6] Saner F.H., Kirchner C. (2016). Monitoring and Treatment of Coagulation Disorders in End-Stage Liver Disease. Visc. Med..

[7] Flores A.S., Forkin K.T., Brennan M.M., Kumar S.S., Winegar D.A., Viola F. (2023). Multicenter evaluation of the Quantra with the QStat Cartridge in adult patients undergoing liver transplantation. Liver Transpl..

[8] Ferrante E.A., Blasier K.R., Givens T.B., Lloyd C.A., Fischer T.J., Viola F. (2016). A Novel Device for the Evaluation of Hemostatic Function in Critical Care Settings. Anesth. Analg..

